# Optically Measured Microvascular Blood Flow Contrast of Malignant Breast Tumors

**DOI:** 10.1371/journal.pone.0099683

**Published:** 2014-06-26

**Authors:** Regine Choe, Mary E. Putt, Peter M. Carlile, Turgut Durduran, Joseph M. Giammarco, David R. Busch, Ki Won Jung, Brian J. Czerniecki, Julia Tchou, Michael D. Feldman, Carolyn Mies, Mark A. Rosen, Mitchell D. Schnall, Angela DeMichele, Arjun G. Yodh

**Affiliations:** 1 Department of Biomedical Engineering, University of Rochester, Rochester, New York, United States of America; 2 Department of Biostatistics & Epidemiology, University of Pennsylvania, Philadelphia, Pennsylvania, United States of America; 3 ICFO- Institut de Ciències Fotòniques, Castelldefels (Barcelona), Spain; 4 Department of Astronomy & Physics, Eastern University, St. Davids, Pennsylvania, United States of America; 5 Department of Physics & Astronomy, University of Pennsylvania, Philadelphia, Pennsylvania, United States of America; 6 The Children's Hospital of Philadelphia, Philadelphia, Pennsylvania, United States of America; 7 Department of Surgery, Hospital of the University of Pennsylvania, Philadelphia, Pennsylvania, United States of America; 8 Department of Pathology and Laboratory Medicine, Hospital of the University of Pennsylvania, Philadelphia, Pennsylvania, United States of America; 9 Department of Radiology, Hospital of the University of Pennsylvania, Philadelphia, Pennsylvania, United States of America; 10 Department of Medicine (Hematology/Oncology), Hospital of the University of Pennsylvania, Philadelphia, Pennsylvania, United States of America; University of Pécs Medical School, Hungary

## Abstract

Microvascular blood flow contrast is an important hemodynamic and metabolic parameter with potential to enhance *in vivo* breast cancer detection and therapy monitoring. Here we report on non-invasive line-scan measurements of malignant breast tumors with a hand-held optical probe in the remission geometry. The probe employs diffuse correlation spectroscopy (DCS), a near-infrared optical method that quantifies deep tissue microvascular blood flow. Tumor-to-normal perfusion ratios are derived from thirty-two human subjects. Mean (95% confidence interval) tumor-to-normal ratio using surrounding normal tissue was 2.25 (1.92–2.63); tumor-to-normal ratio using normal tissues at the corresponding tumor location in the contralateral breast was 2.27 (1.94–2.66), and using normal tissue in the contralateral breast was 2.27 (1.90–2.70). Thus, the mean tumor-to-normal ratios were significantly different from unity irrespective of the normal tissue chosen, implying that tumors have significantly higher blood flow than normal tissues. Therefore, the study demonstrates existence of breast cancer contrast in blood flow measured by DCS. The new, optically accessible cancer contrast holds potential for cancer detection and therapy monitoring applications, and it is likely to be especially useful when combined with diffuse optical spectroscopy/tomography.

## Introduction

Breast cancer is the leading cause of cancer death among women worldwide [1], and advances in early detection, accurate diagnosis, and prediction of therapeutic efficacy are important for improving the survival of those affected by the disease [2]. To this end, development of new techniques which complement information provided by routine clinical imaging methods is desirable.

Diffuse optical spectroscopy (DOS) and tomography (DOT) are relatively new non-invasive and low-cost techniques that provide unique functional information for breast cancer applications using near-infrared (NIR: 650–1000 nm) light sources [3]. NIR light penetrates up to 10 cm in breast tissue, and the use of the photon diffusion equation in signal analysis permits decoupling of tissue optical absorption from scattering. Thus, DOS and DOT measurements provide quantitative information about tissue total hemoglobin concentration, blood oxygenation and scattering parameters, which are not accessible by mammograms and ultrasonograms. To date, breast cancer studies with DOS and DOT have found endogenous total hemoglobin concentration to be higher in malignant tumors compared to surrounding healthy tissue and benign tumors [3]–[23]; further, varied reports exist for contrast in other functional optical parameters [3], [4], [24]. Recently, recognition of the suitability of DOS/DOT for frequent bedside monitoring has led to new investigations of the utility of the technology for neoadjuvant (pre-surgical) chemotherapy monitoring. Indeed, DOS/DOT has exhibited sensitivity to changes induced by breast cancer therapies and has demonstrated potential to predict therapeutic efficacy [25]–[44].

One limitation of DOS/DOT for early cancer detection is its relatively low image resolution. To overcome this limitation, new types of tumor contrast are being actively explored, including exogenous absorption/fluorescence contrast agents [45]–[48] and the use of perturbations to induce tumor-sensitive hemodynamic changes [49]–[52]. These new tumor contrast measurements that are not present in standard clinical images have the potential to improve tumor detection, characterization and therapeutic efficacy prediction.

Blood flow is a driver for tumor tissue metabolism and oxygenation which, in turn, affects the efficacy of several cancer therapies [53]. Very recently, blood flow changes in breast cancer patients undergoing neoadjuvant chemotherapy were shown to predict disease-free and overall survival [54]. As a result, breast cancer blood flow has drawn clinical interest and has been quantified using 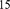 O-water Positron emission tomography (PET) [54]–[60], 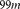 Tc-Sestamibi with Single-photon emission computed tomography (SPECT) [61]–[63], dynamic 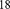 F-FDG-PET [54], [64], dynamic contrast-enhanced Magnetic Resonance Imaging (DCE-MRI) using kinetic modeling or a deconvolution technique [65], [66], Arterial Spin Labeling MRI [67] and color/power Doppler ultrasound [68]–[73]. However, none of these methods is ideal for quantifying microvascular blood flow. For example, PET and SPECT require the injection of radioactive contrast agent; MRI measurements are expensive, and in addition, clinical diagnostic MRI requires contrast agent injection [74]; Doppler ultrasound is inexpensive and can measure blood flow in large vessels without a contrast agent, but ultrasound measurement of microvascular blood flow requires injection of an exogenous contrast agent [72].

Here we examine the utility of a relatively new optical technique, diffuse correlation spectroscopy (DCS), for measurement of microvascular blood flow in breast tumors and healthy breast tissues. The DCS method employs the temporal fluctuations of near-infrared light intensity to measure blood perfusion without the injection of a contrast agent [3]. The technique is non-invasive and utilizes relatively inexpensive equipment. Previously, a preliminary study using DCS to measure blood flow in human breasts showed increased blood flow contrast in tumor regions relative to adjacent healthy tissue (3 subjects with malignant tumors, 2 subjects with benign tumors, 2 healthy subjects) [75]. In addition, several pilot studies have suggested that DCS can track temporal changes in microvascular blood flow induced by neoadjuvant chemotherapy [26], [33] and targeted therapy [26]. While the results of these case studies are promising, the work thus far has been carried out with very few patients, and more data are required to quantify blood flow contrast in breast cancer. In this contribution, we report the ratio of DCS-measured microvascular blood flow in the tumor-versus-normal tissues of 32 patients with malignant breast tumors. On average, a statistically significant two-fold increase in blood flow in the tumor was observed when compared either to surrounding normal tissue or to contralateral (normal) tissue. To the best of our knowledge, this study is the first where DCS-derived blood flow contrasts of malignant breast tumors were systematically quantified in a large patient cohort. In addition, we examine the choice of normal tissue in terms of quantifying tumor-to-normal contrast. These findings represent an important first step towards assessment of tumor blood flow accessible to optical methods, and in particular, whether this contrast can enhance breast cancer detectability, diagnosis, and therapy monitoring.

## Materials and Methods

### DCS Instrumentation

**Figure 1:**
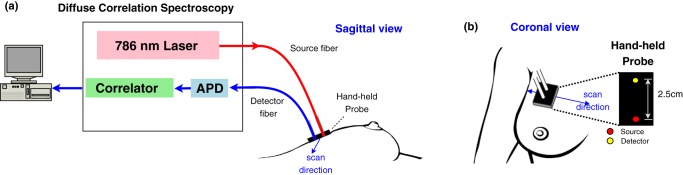
Instrument and probe placement. (a) Diagram of diffuse correlation spectroscopy and probe placement on a breast in sagittal view. Near-infrared light from a 786 nm long coherence laser is delivered to the breast surface via a multi-mode optical fiber (source fiber) attached to a hand-held probe. A single-mode optical fiber (detector fiber) attached to the hand-held probe collects and relays the light signal to a photon-counting avalanche photodiode (APD). An autocorrelator board calculates normalized temporal intensity autocorrelation functions of the detected light and passes the functions onto the computer for further postprocessing. (b) Schematic of probe configuration and its placement on a breast in coronal view. A source-detector separation of 2.5 cm was used in this study.

A custom-built DCS instrument consisting of a 786 nm long-coherence-length laser (CrystaLaser, Reno, NV), fast photon-counting avalanche photodiodes (Excelitas, Waltham, MA) and a correlator board (Correlator.com, Bridgewater, NJ) was utilized to record temporal intensity autocorrelation function. Details of the instrument can be found in References [3], [76]. A schematic of the instrument is shown in Figure 1(a), and the probe design/placement on tissue is shown in Figure 1(b). Laser light was delivered onto the breast tissue surface via a multi-mode fiber. A single-mode fiber was utilized for detection of light that traveled through the breast tissue. The source and detector fibers were separated by 2.5 cm on the tissue surface.

### Ethics Statement

The study was conducted according to a protocol approved by the University of Pennsylvania Institutional Review Board. Written informed consent was obtained from each subject prior to diffuse optical measurements.

### Human Subject Measurement Protocol

Female patients previously diagnosed with malignant breast tumors by biopsy were recruited. For diffuse optical measurements, the subject lay on a table in a supine position. First, the tumor location was identified either by palpation or consulting radiology reports from previous imaging, and the tumor center was marked with a skin-compatible marker on the cancerous breast. Then, an additional 10–12 positions (1 cm apart) were marked on a line straddling the tumor. This line was chosen to include both cancer and normal tissue. For data collection, the probe was placed gently on the breast tissue, and five DCS measurements were acquired at each position. Subsequently, measurements were taken in similar manner on the contralateral breast. The measurement protocol for the contralateral breast evolved over time. For the first 9 subjects, contralateral breast measurements were performed at a spatial position whose location corresponded to the tumor center position reflected about a sagittal plane through the chest center. Hereafter, we will denote this location as “mirror-image position”. For the remaining 23 subjects, contralateral breast measurements were performed at 11–13 positions, corresponding to the mirror-image of the “measurement line” in the ipsilateral cancerous breast.

### Data Analysis

#### Extraction of blood flow index

To derive the tissue blood flow index, 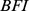, the measured DCS temporal light intensity autocorrelation functions were fit to a solution of the correlation diffusion equation in the homogeneous semi-infinite geometry [3]. At each spatial position, five 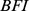 values were averaged. The solution depends on the tissue absorption and reduced scattering coefficients 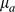 and 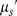 at 786 nm. In this study, these parameters were assumed to be 0.05 and 8 cm^-1^ respectively, based on literature values [77] and previous data [20]. These coefficients were measured with DOS in less than 50% of the subjects, due to lack of concurrent DOS instrumentation in the probe; therefore, for consistency we chose to utilize assumed absorption and scattering parameters. The effect of this assumption about tissue absorption and scattering was investigated further (see Results), and we do not believe that the observations we report are altered significantly as a result of this assumption.

#### Selection of regions to quantify regional blood flow

**Figure 2:**
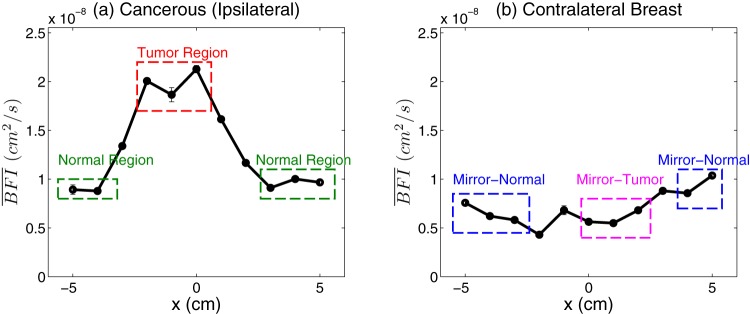
Selection of regions to quantify regional blood flow. Line-scans of 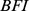 is shown (a) for ipsilateral cancerous breast and (b) for contralateral breast respectively. First, tumor (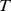) and normal (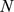) regions are chosen in the ipsilateral breast. Then the mirror positions of tumor (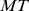) and normal (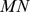) regions are identified in the contralateral breast.

Typical line-scans of ipsilateral tumor-bearing and contralateral breasts are shown in Figure 2. 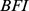 at the center of cancerous breast was elevated compared to that of surrounding normal regions. In this population, the 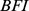 of contralateral breasts showed less spatial variation than that of cancerous breasts. The choice of tumor region (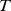) was guided by the tumor location from radiology report/images and the local 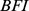 peak. Normal regions (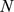) were chosen such that they were far from the tumor region and were not near hemodynamically abnormal tissue (e.g., biopsy-induced bleeding or scar). Since the measurements were performed with an *a priori* knowledge of approximate tumor location, most of tumor regions were found to be located around the center of the line (i.e., zero position), and the normal regions were located near both ends of the line segment. For the contralateral breast, mirror positions of the tumor region and the normal region were located and designated as 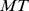 and 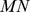 respectively. Within each region, the mean 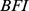 values were calculated as 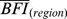 where 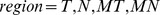 for the tumor (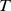) and normal (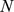) tissues in the ipsilateral cancerous breast, and for the mirror positions of the tumor (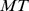) and normal (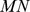) tissues in the contralateral breast.

#### Quantification of relative blood flow

We collected data from normal tissue in both the ipsilateral (cancer-bearing) and contralateral breasts, permitting various definitions of relative blood flow. The definitions of five different types of blood flow analysis are listed in the Table 1. From the ipsilateral measurements, 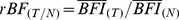 was available for all subjects. In addition, 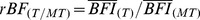 was available for all subjects because every study protocol included the measurements at the mirror position of the tumor in the contralateral breast. Other types of relative blood flow, such as 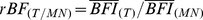, 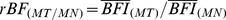 and 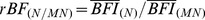, were available for a subset of the subjects (i.e., n = 23).

**Table 1:** Definition of relative blood flows based on local regions.

Parameter	Definition	Comments
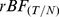	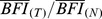	tumor vs. normal in ipsilateral breast
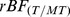	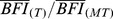	tumor vs. its mirror position
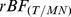	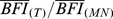	tumor vs. mirror position of normal
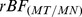	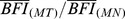	mirror position tumor vs. normal in contralateral breast
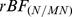	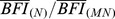	normal vs. its mirror position

#### Data exclusion criteria

In this study, 42 patients who were previously diagnosed with malignant breast tumors via biopsy were measured during a six year time period from 2004–2010. Data from 10 out of the 42 patients were excluded from the final analysis presented in this report for the following reasons: Five had a complicated clinical history such as previous surgery, breast implant, multiple lesions, or biopsy-induced bleeding which yielded a prohibitively low DCS signal; five had incomplete or unreliable DCS measurements due to limited time available for the contralateral measurements or difficulty in probe contact.

### Statistical Analysis

Data from individual regions were summarized using means and standard deviations. To assess differences in the mean 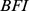 levels (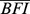) between regions, we log-transformed the data to achieve approximate normality, and we constructed a mixed effects model either with individual regions or with breast side (contralateral versus ipsilateral) as the predictor. Mixed effects models are a type of linear regression that allow repeated, and hence correlated, measurements on individual subjects [78]. Once the model was fit we carried out a series of two-sided tests of differences in 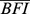. Specifically we tested for overall differences in mean 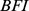 between the contralateral and ipsilateral breasts and for differences between the tumor versus normal region of the ipsilateral breast, or the mirror tumor or normal region of the contralateral breast. We also determined whether there was evidence that the difference in the log of 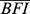 between the tumor and normal region of the ipsilateral region differed from the difference in log of 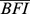 between the tumor and normal region of the contralateral breast. Estimates, and 95% confidence intervals (CI) for the mean ratio of 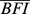 in the different regions were constructed by exponentiating the relevant terms from the model. The type I error rate was set to 0.05. Lastly we determined the percent (and its 95% CI) of all individuals for which 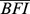 in the region of interest (e.g., tumor or ipsilateral breast) exceeded that in a contrasting region (e.g., normal, mirror positions, or contralateral breast). Analyses that did not include the mirror normal (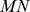) region included all subjects (n = 32), while analyses that included the mirror region used only 23 subjects with measurements in all regions. All analyses were carried out in R (version 3.02).

## Results

### Subject Characteristics

Thirty-two female subjects with biopsy-proven malignant lesions were included in this analysis. Demographic, radiologic, and histopathologic information for these subjects are presented in Table 2. In addition, information on the time of optical measurement in relation to core biopsy are presented. Subset 1 refers to the first 9 subjects with the availability of contralateral breast measurements at only one position, and subset 2 refers to the remaining 23 subjects with contralateral breast measurements at 11–13 positions.

**Table 2:** Clinical characteristics of subjects.

Parameters	subset 1	subset 2	whole set
Number of subjects	9	23	32
Age (yr)	51±8	50±11	50±10
BMI (kg/m^2^)	29.1±8.2	27.3±5.8	27.8±6.5
Menopausal status			
Premenopausal	3 (33%)	14 (61%)	17 (53%)
Postmenopausal	5 (56%)	7 (30%)	12 (38%)
Perimenopausal	1 (11%)	1 (4%)	2 (6%)
Unknown	0 (0%)	1 (4%)	1 (3%)
Race			
Caucasian	9 (100%)	19 (83%)	28 (88%)
African American	0 (0%)	4 (17%)	4 (12%)
Mammographic density			
Almost entirely fat	0 (0%)	1 (4%)	1 (3%)
Scattered fibroglandular densities	3 (33%)	7 (30%)	10 (31%)
Heterogeneously dense	4 (44%)	12 (52%)	16 (50%)
Extremely dense	0 (0%)	1 (4%)	1 (3%)
Unknown	2 (22%)	2 (9%)	4 (13%)
Lesion Type (primary component)			
Invasive ductal carcinoma	8 (89%)	20 (87%)	28 (88%)
Invasive lobular carcinoma	0 (0%)	2 (9%)	2 (6%)
Ductal carcinoma *in situ*	1 (11%)	1 (4%)	2 (6%)
Maximum Lesion dimension (cm)	3.0±1.2	5.5±3.4	4.8±3.2
Estrogen Receptor (ER)			
ER+	4 (44%)	15 (65%)	19 (59%)
ER−	5 (56%)	8 (35%)	13 (41%)
Progesterone Receptor (PR)			
PR+	5 (56%)	12 (52%)	17 (53%)
PR−	4 (44%)	11 (48%)	15 (47%)
Human epidermal growth factor receptor 2 (HER2)			
HER2/neu+	3 (33%)	5 (22%)	8 (25%)
HER2/neu−	6 (67%)	18 (78%)	24 (75%)
Optical measurement with respect to core biopsy			
Before any core biopsy	2 (22%)	4 (17%)	6 (19%)
<14 days after core biopsy	0 (0%)	5 (22%)	5 (16%)
≥14 days after core biopsy	7 (78%)	14 (61%)	21 (65%)

For continuous data such as age and body mass index (BMI), mean 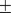 standard deviations are shown. For each categorical variable, the number of women is given and the percentage of the total number of women in the group appears in parentheses.

Subject characteristics of all 32 subjects (whole set) in this study are summarized in the following text. No patient had more than one lesion. The mean lesion size along its longest dimension was 4.8 cm, and it ranged from 0.8 to 13.8 cm across the patient cohort. A majority of women were premenopausal (53%) and Caucasian (88%), and the patient population had an average age of 50 years. The mean body mass index was in the overweight category by World Health Organization criteria [79]. More than half of subjects with known mammographic density had heterogeneously dense or extremely dense breasts as determined by X-ray mammography. Unlike X-ray mammography, DCS is not limited by high radiographic breast density; thus, successful DCS measurements were carried out in this population which was comprised of more than 50% premenopausal women, as well as women with radiographically dense breasts. Characteristics of subset 2 and of subset 1 were similar, except that subset 1 had a smaller proportion of pre-menopausal women (33% vs 61%) and a smaller mean size (3.0 vs 5.5 cm).

Most subject characteristics were similar to our previous study using DOT [20], except that the average lesion size was somewhat larger in the present study (i.e., Present study: 4.8
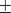
3.2 cm; [20]: 2.1
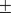
1.2 cm). This difference is due to inclusion of subjects with locally advanced breast cancer (i.e., malignant lesions larger than 5 cm).

### Blood Flow Tumor Contrast

**Figure 3:**
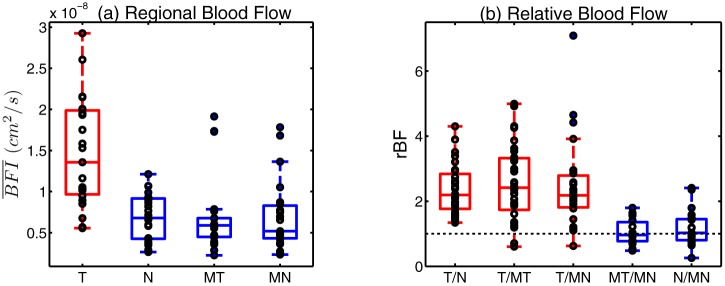
Blood flow contrast. (a) Boxplot of average blood flow (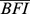) per region: 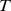 refers to the tumor region (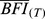) and 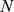 refers to the normal region (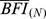) in the ipsilateral breast. 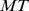 refers to the mirror tumor region (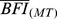) and 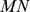 refers to the mirror normal region (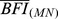) in the contralateral breast. (b) Boxplot of relative blood flow (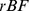): 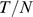 refers to the blood flow ratio between the tumor region and the normal region in the ipsilateral breast (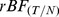), 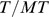 refers to that between the tumor region and the mirror tumor region (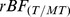), and 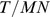 refers to that between the tumor region and the mirror normal region (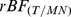). 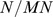 refers to the blood flow ratio between the normal region in the ipsilateral breast and the mirror normal region in the contralateral breast (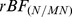), and 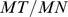 refers to that between the mirror tumor region and the mirror normal region in the contralateral breast (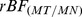). Each circle corresponds to an individual data point.

**Figure 4:**
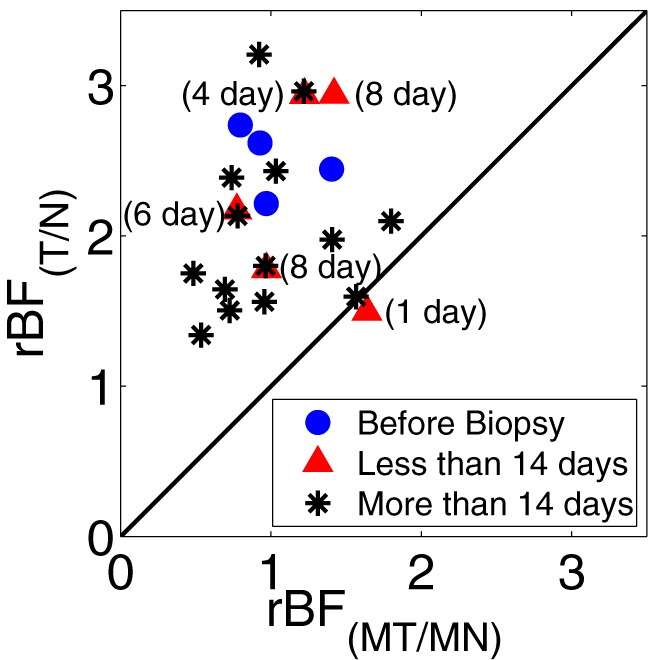
Difference between relative blood flow from ipsilateral and contralateral breast. 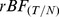
 from the ipsilateral breast versus 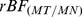 from the contralateral breast. Optical measurements were performed before any core biopsy (blue solid circle), at less than 14 days (red solid triangle) or at more than 14 days after core biopsy (black asterisk).

Figure 3(a) shows boxplots of 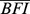 in the tumor and in each of the normal regions (normal ipsilateral, and in the mirror sites at the contralateral breast). The mean and standard deviation in tumor regions are 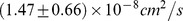, while in normal regions these values are 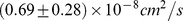, 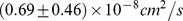, 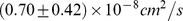 for regions 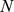, 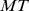, and 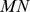 respectively. These results suggest that the tumor region tended to have higher 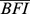 values, both on average and individually, than the normal regions. Figure 3(b) shows boxplots of three kinds of tumor-to-normal 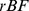 values (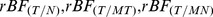) and two kinds of normal-to-mirror normal 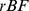 values (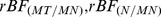). These plots show that the individual values for each of the tumor to normal regions tend to fall above 1.0 while those for the paired normal regions tend to be centered around 1.0. Additionally Figure 4 shows the tumor-to-normal 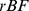 for ipsilateral breast versus mirror tumor-to-mirror normal 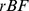 for contralateral breast. Figure 4 confirms that the 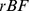 for the tumor to normal region of the ipsilateral breast typically exceeds that of the mirror region in the contralateral breast. Note that we divided the data into three groups based on the delay between biopsy and optical measurement and explicitly indicated those with a core biopsy less than two weeks prior to the measurement in Figure 4. This distinction using 14 days is based on the observation from a prior optical study using diffuse optical spectroscopy on breast [80]. In particular, Tanamai *et al.* tracked the changes in physiological parameters before and after 9, 17, 23, 30, 37, 44, 51, and 58 days after a core biopsy on a single subject with fibroadenoma. They noted significant elevation of the tissue optical index (TOI  =  deoxyhemoglobin 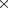 water/lipid) with respect to the pre-biopsy baseline at days 9 and 17, and concluded that a minimum of 14 days post-biopsy was required to return TOI to baseline value [80]. In our study, there were several subjects measured within fourteen days of core biopsies, and measurements were not significantly different from the remaining subjects. Table 3 shows that the mean 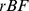 for the tumor versus normal values for any of the three normal regions was between 2.25 and 2.27, and that the 95% CI for the three ratios were similar. In all three cases, the mean 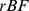 for the tumor-to-normal region was significantly greater than 1.0 (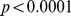). In contrast, the mean 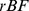 for the normal regions, either the mirror tumor-to-mirror normal in the contralateral breast or the normal-to-mirror normal region was essentially 1.0. Lastly 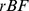 constructed based on the four regions was 2.25, a value that was again significantly greater than 1.0 (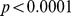).

**Table 3:** Relative blood flow parameters based on different regions.

Parameter	mean (95% CI)	*p*	*n*
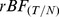	2.25 (1.92–2.63)	<0.0001	32
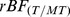	2.27 (1.94–2.66)	<0.0001	32
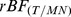	2.27 (1.90–2.70)	<0.0001	23
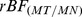	1.00 (0.84–1.19)	0.97	23
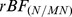	1.01 (0.85–1.20)	0.92	23
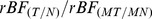	2.25 (1.78–2.85)	<0.0001	23

Mean (95% confidence interval) of relative blood flow based on different regions and 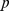 values testing the hypothesis that 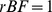. (See Table 1 for definitions of each parameter.) 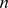 is the number of subjects used for calculating corresponding 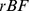 value.

Table 4 shows the proportion of subjects, and associated 95% CI, for whom the individual 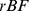, constructed using the different normal values, exceeded 1.0. These proportions ranged from 94 to 100% for the tumor-to-normal regions and 39 to 57% for the paired normal regions. These values suggest that the method has the potential for high sensitivity for detection/confirmation of malignant tissue.

**Table 4:** Proportion of individuals with relative blood flow parameters higher than 1.

Parameter	Percentage (95% CI)	*n*
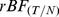	100 (89–100)	32
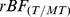	94 (79–99)	32
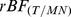	96 (78–100)	23
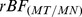	39 (20–61)	23
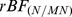	57 (34–77)	23
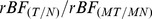	100 (85–100)	23

Percentage (%) and its 95% confidence interval are listed for each relative blood flow parameter. (See Table 1 for definitions of each parameter.) 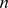 is the number of subjects used for calculating corresponding 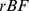 value.

#### Effect of DOS-derived optical properties in quantification of 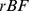


**Figure 5:**
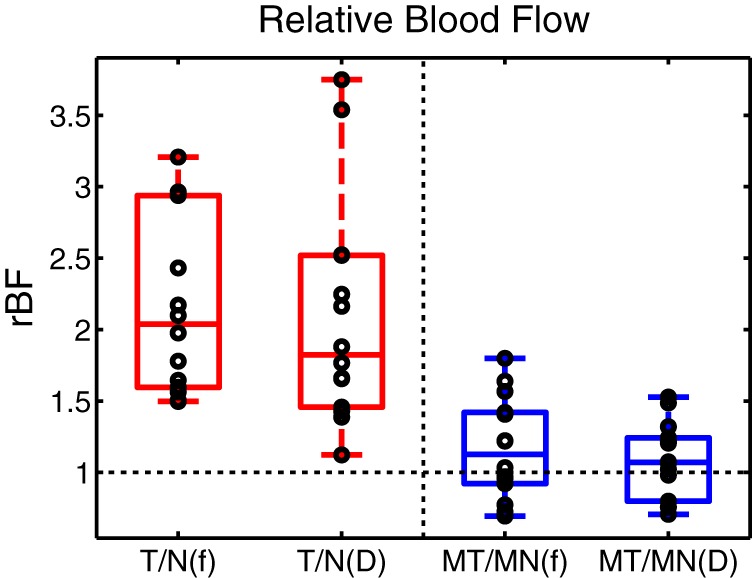
Effect of DOS-derived optical properties on 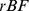. 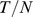
 refers to the blood flow ratio between the tumor region and the normal region in the ipsilateral breast (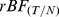). 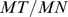 refers to that between the mirror tumor region and the mirror normal region in the contralateral breast (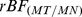). 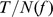 and 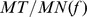 were computed using assumed fixed optical properties to quantify 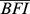 values for 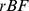. This analysis method was utilized throughout the study. 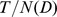 and 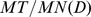 were computed using DOS-derived optical properties in quantifying 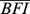 values.

Out of 32 subjects, 14 subjects were measured using a hybrid instrument combining both DOS and DCS units into a single non-invasive probe. The DOS unit was based on 70 MHz homodyne frequency-domain system with three different wavelengths (675, 786, 830 nm) and an avalanche photodiode detector [81]. After all tissue measurements, calibration measurements were performed on a liquid tissue phantom with known optical properties (e.g., 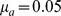 cm^-1^ and 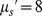 cm^-1^ at 786 nm).


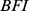
 s were calculated using the solution to correlation diffusion equation for a homogeneous semi-infinite medium [3], with or without DOS-derived optical properties. In Figure 5, 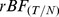 and 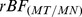 from fixed (f) optical properties and DOS-derived (D) optical properties are presented. When 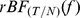 and 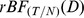 were compared using two-tailed two-sample t-test, they were not significantly different from each other. Additionally, the difference between the 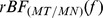 and 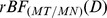 was not significant either.

## Discussion

The primary goal of this study was to quantify tumor-to-normal contrast of local, microvascular blood flow using diffuse correlation spectroscopy measurements in a large patient cohort. To date, many investigations have quantified breast tumor-to-normal contrast of hemoglobin concentrations and tissue absorption/scattering parameters using diffuse optical spectroscopy and imaging [4]. However, blood flow contrast has not been investigated to a similar degree. Carp *et al.* developed a technique to extract blood flow based on oxygenated and deoxygenated hemoglobin concentrations from DOS measurements [82]. However, this method requires breast compression, rapid data acquisition of transient dynamics, and is an indirect measure of blood flow based on several assumptions. DCS is a simple steady-state optical method that extracts blood flow indices of underlying tissues directly.

In a preliminary study of breast cancer blood flow contrast with DCS [75], we introduced the method and reported 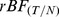 for 7 subjects (3 subjects with a malignant tumor). In the present work, DCS-derived blood flow contrast is characterized in 32 subjects with known malignant tumors, and the blood flow relationship between ipsilateral and contralateral breasts is also studied. Tumor regions had statistically significant higher flow contrast compared to normal tissue regions and their counterparts in the contralateral breast. This finding is consistent with results from ultrasound [68], [70], [72], positron emission tomography [57], and magnetic resonance imaging [65]. Note that the units of the DCS-derived blood flow index 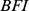 (cm^2^/s) do not match those used in the clinic (ml/min/100ml of tissue); thus, direct comparison between DCS and other modalities using absolute values will require more calibration and is not yet practical. In the future, DCS-derived 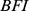 can be calibrated to yield absolute values via concurrent comparison with flow tracer measurements [83], [84]. Note, however, good correlations have already been found between relative blood flow measured by DCS and relative blood flow measured by the various other imaging modalities such as Xenon-CT, ASL-MRI, and Doppler ultrasound [3]. In the present work, the average blood flow ratio measured by DCS between tumor and normal tissue within ipsilateral breasts (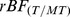) was 2.25, and between tumors and mirror sites in contralateral breasts was 2.27. The reported average 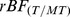 measured by 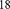 O-PET varied from 3.6 to 5.2 [55], [57], [85], [86], and the reported average 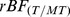 and 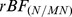 measured by deconvolution MRI ranged around 5.1 and 1.1 respectively [65]. Note that difference between ipsilateral and contralateral normal regions quantified by 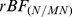 with DCS (1.0) and with MRI (1.1) are roughly equivalent. The DCS measurement of 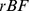 thus had the same sign as observed by other techniques, albeit a somewhat lower magnitude.

The smaller tumor-to-normal contrast observed by DCS may be due to partial volume effects. Due to limited number of sources and detectors, our data analysis assumed the probed tissue was a homogeneous semi-infinite medium. Partial volume effects are more pronounced in the remission geometry compared to transmission geometries, and these effects could produce an underestimation of blood flow. Importantly, however, the two-fold blood contrast observed in the present *remission* study is significantly higher than relative total hemoglobin concentration (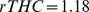) or reduced scattering coefficient contrast (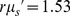) observed in our previous *transmission* study [20]. Another limitation of the study is the use of fixed literature values for optical properties in the data analysis, i.e., rather than using the optical properties (absorption and reduced scattering coefficients) measured independently for each individual subject. Note that we have carried out concurrent DOS measurements in fourteen of the thirty-two subjects, and we investigated the effects of incorporating these data into DCS analysis (see Results). While 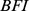 and 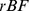 values of individual subjects can change with the incorporation of more accurate spatial variation of optical properties, the overall conclusion of this study (i.e., tumor regions have higher blood flow compared to normal tissues of the ipsilateral and contralateral breast) was not significantly affected. Nevertheless, future concurrent measurements of individual optical properties with DOS [3] would not only improve the accuracy of the 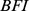 measurement, but also provide a more complete picture of the tumor physiology and metabolism through tumor blood volume, blood oxygenation, and water content. This approach of concurrent DOS and DCS measurements is useful for improving specificity and therapy efficacy predictions [87].

In designing measurement protocol for diffuse optical techniques, one may also decide to limit the data collection from the contralateral breast depending on the time or data normalization strategy. Usually this type of limitation is justified under the assumptions either that hemodynamic parameters of ipsilateral normal regions and contralateral regions are similar, or that the spatial variation of hemodynamic parameters in the contralateral breast is minimal. Our study shows that these assumptions are reasonable most of the time (e.g., 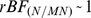), but that exceptions sometimes arise in some individuals (e.g., 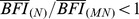. Ultimately, these exceptions may be important to detect, since the overall blood flow discrepancy in different breasts may be related to the effectiveness of chemotherapeutic delivery [88].

Since diffuse optical techniques are sensitive to hemodynamic changes, there is a concern for the potential contamination of the optical signal due to inflammation and/or post-procedural bleeding induced by the biopsy procedure. While we used 14 days post-biopsy as the threshold for a potential biopsy effect based on Reference [80], we note that changes detected by DOS in that reference was mainly from water content and not from oxygenated or deoxygenated hemoglobin concentrations at day 9. Even though we cannot separate the wound-healing effect due to core biopsy, when we categorized our data with respect to days after biopsy, we did not see any noticeable deviation of the subset of subjects measured within 14 days post-biopsy from the rest of subjects. Further detailed studies are warranted to quantify the effect of wound healing in breasts by making concurrent DOS and DCS measurements on multiple subjects before and after core biopsy longitudinally over at least two weeks with shorter time intervals (i.e., daily or semi-daily intervals).

In future studies, partial volume effects may be minimized through optimization of probe and incorporation of depth information (e.g., from ultrasound or other imaging input). In addition, the weaknesses of the remission geometry measurement can be overcome by switching to the transmission geometry. Recently, Busch *et al.* demonstrated transmission DCS measurements in the breast [89]. However, achieving an adequate signal-to-noise ratio is challenging in the transmission geometry which limits data acquisition rates due to increased (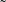
5 cm) separations between sources and detectors [89]. In addition, a successful combination of diffuse optical spectroscopy and diffuse correlation spectroscopy with the capability to overcome partial volume effect through more involved modeling (e.g., tomography) can offer an enabling tool for investigation of breast cancer oxygen metabolism. With better quantification and with more subjects, it will also be interesting to search for significant differences in blood flow contrast due to tumor characteristics connected with estrogen receptor (ER), progesterone receptor (PR), and human epidermal growth factor receptor 2 (HER2/neu) [90], tumor grade, and tumor size.

## Conclusion

We have shown that blood flow measurements using diffuse correlation spectroscopy in the remission geometry differentiate breast cancer from surrounding normal tissue and/or contralateral normal breast tissue, with two-fold contrast. These encouraging results suggest that blood flow is a useful and readily measurable hemodynamic biomarker with the ability to differentiate malignant tumors from healthy tissue via non-invasive diffuse optical methods. In the future, we also expect that the combination of all available hemodynamic parameters will improve breast cancer detection and therapy monitoring/prediction.
